# Dysregulation of iron metabolism modulators in virologically suppressed HIV-infected patients

**DOI:** 10.3389/fimmu.2022.977316

**Published:** 2022-11-25

**Authors:** Vanesa Garrido-Rodríguez, Ana Isabel Álvarez-Ríos, Israel Olivas-Martínez, María del Mar Pozo-Balado, Ángel Bulnes-Ramos, Manuel Leal, Yolanda María Pacheco

**Affiliations:** ^1^ Immunology Service, Institute of Biomedicine of Seville, Institute of Biomedicine of Seville (IBiS), Virgen del Rocío University Hospital/CSIC/University, Seville, Spain; ^2^ Biochemistry Department, Virgen del Rocío University Hospital, Seville, Spain; ^3^ Internal Medicine Service, Hospital Viamed, Santa Ángela de la Cruz, Seville, Spain; ^4^ Medical Service, Santa Caridad Home for the Elderly, Seville, Spain

**Keywords:** chronic HIV infection, iron metabolism, soluble transferrin receptor, hepcidin, inflammation

## Abstract

**Background:**

Iron metabolism plays an essential role in cellular functions. Since virologically suppressed chronic HIV-infected subjects under effective antiretroviral treatment (ART) exhibit a persistent immune dysfunction that leads to comorbidities, iron homeostasis may be relevant in this context. We aimed to explore iron metabolism in virologically suppressed chronic HIV infected subjects under a successful ART.

**Methods:**

In this retrospective study, traditional iron metabolism biomarkers (total iron, ferritin, transferrin, and transferrin saturation index), as well as soluble transferrin receptor (sTfR), hepcidin, and inflammatory markers were determined in virologically suppressed chronic HIV-infected subjects under at least 2 years of ART (HIV) who also had >350 CD4-T-cells/mm^3^ (N=92) from Spain. As controls, we collected non-HIV age-matched healthy donors (Young, N=25) and elderly subjects (>65 years old; Elderly; N=25). Additionally, an external group of non-HIV patients with ferritin<50 ng/mL diagnosed with absolute iron deficiency (Ferropenic group; N=84) was included. Comparisons between groups were performed using Kruskal-Wallis or Mann-Whitney U-tests, while associations between variables were explored by Spearman’s rho correlation coefficient.

**Results:**

We selected samples from HIV-infected subjects (aged 42[34-47], 95% males), young age-matched (aged 40[30-58], 60% males), and elderly controls (aged 82[78-88], 100% males). Compared to both healthy (Young and Elderly) groups, HIV exhibited decreased iron, transferrin saturation, and sTfR, and increased ferritin, but similar hepcidin levels. Notably, associations between sTfR and iron (Young, *r*=-0.587, *p*=0.002; Elderly, *r*=-0.496, *p*=0.012) or transferrin saturation index (Young, *r*=-0.581, *p*=0.002; Elderly, *r*=-0.489, *p*=0.013) were negative in both controls while positive in HIV (*r*=0.464, *p*<0.0001 and *r*=0.421, *p*<0.0001, respectively). Moreover, the expected negative correlation between hepcidin and sTfR, observed in controls (Young, *r*=-0.533, *p*=0.006; Elderly, *r*=-0.473, *p*=0.017), was absent in HIV (*r*=0.082; *p*=0.438). Interestingly, the HIV inflammatory profile differed from the Elderly one, who despite their inflammaging-related profile, succeed in maintaining these associations. Furthermore, subjects from the ferropenic group (aged 42[32-51], 5% males), showing significantly lower levels of hepcidin and higher sTfR, as expected, reflected similar correlations as those Young and Elderly, in contrast to HIV.

**Conclusions:**

Virologically suppressed chronic HIV-infected patients under successful ART exhibit altered levels of iron metabolism modulators suggesting a complex functional iron deficiency.

## Introduction

Subjects with chronic HIV infection present a profound immune dysfunction as well as a persistent low-grade inflammation, despite effective antiretroviral treatment (ART), which underlies their clinical progression with a higher risk of comorbidities and mortality (1). Iron plays a crucial role in immune cell function and host defense; therefore, its regulation may be relevant in chronic HIV infection. Innate immune responses such as macrophage polarization rely on iron availability (2). Regarding adaptive responses, many metabolic and redox reactions involved in T-cell effector functions, cell division, and cytokine production are iron-dependent whereas transferrin receptor CD71 mediates the activation and proliferation of T-cells (3).

Iron is transported in plasma bound to transferrin, while is intracellularly stored bound to ferritin. The iron export from cell to blood is mediated by ferroportin, a transporter located mainly in the membrane of enterocytes and macrophages. Under physiological conditions, the pool of transferrin is about 30% saturated with iron and supplies iron to all cells by binding to transferrin receptors CD71 (TfR) (4). The regulation of iron levels is tightly controlled, the hormone hepcidin being the main regulator of this complex system (5). When plasma iron/transferrin saturation increases, hepcidin is secreted by hepatocytes and binds ferroportin, which is internalized and degraded, resulting in a decreased iron export from enterocytes to blood and an accumulation in macrophages in the spleen and liver (6). In contrast, when plasma iron/transferrin saturation decreases, hepcidin transcription is blocked, allowing iron to be released through ferroportin. Hepcidin secretion is also suppressed during erythropoietic demand (via erythroferrone-mediated BMP6 suppression) (7). Cellular surface TfR1 is also upregulated as a mechanism to acquire plasma iron and typically associates with increased cellular iron demand, particularly erythropoietic demand, due to the increased TfR1 expression on erythroblasts (4). A soluble truncated form of isoform 1 of TfR is also found in plasma (sTfR) and reflects total TfR concentration (7).

Interestingly, infections can trigger hepcidin expression through the activation of innate immune responses and the secretion of pro-inflammatory cytokines (e.g. interleukin-6) (8). Regarding HIV infection, iron accumulation and hepcidin can promote HIV transcription and replication, increasing chronic inflammation in an *in vitro* model of infection (9) and hepcidin levels have been inversely associated with CD4 T-cell counts and clinical progression (10).

When iron intake or stores are inadequate to meet the physiological requirements, absolute iron deficiency (so-called ferropenia) is occurring, however, in patients with inflammatory diseases the iron stores could be sufficient but its mobilization/utilization is inadequate, thus occurring a functional iron deficiency (11). In general, iron deficiencies (ID) are the most common cause of anemia, particularly in settings of poor diet quality, infections, or genetic disorders (12), however, these deficiencies do not necessarily involve the development of anemia.

Such alterations in the HIV infection scenario have been mainly studied in patients from low/middle-income countries with malnutrition, opportunistic infections, and/or limited access to treatment who had been mostly diagnosed with anemia (10, 13). Moreover, due to the concurrence of these issues, it is difficult to define the specific nature and causes underlying the alterations in iron metabolism in this context. Therefore, studies addressing the relationships between the regulators of iron metabolism in the absence of anemia have not been carried out so far in virologically suppressed chronic HIV infected patients. Since the iron status and its homeostasis could potentially contribute to the persistent immune dysfunction in successfully-treated chronic HIV-infected subjects, we have explored such homeostasis and the interrelations among different iron-related parameters, including hepcidin and sTfR, which are recently being proposed as effective markers of the iron status.

## Methods

### Subjects of study

In this retrospective study, we used available cryopreserved samples of chronic HIV-infected subjects successfully treated and persistently suppressed (<20 copies/mL) during at least 2 years of ART who, at the moment of sample storage, did not present any active infection or malignancies from two different sources: a) from Virgen del Rocío University Hospital (HUVR) (N=28) and b) from the HIV Biobank integrated into the Spanish AIDS Research Network (N=64) (14). As comparison groups, we included 25 age-matched non-HIV healthy donors (Young, Y) from our institution and 25 non-HIV elderly subjects (>65 years old; Elderly, E) from the “Santa Caridad home for the elderly”. Additionally, an external group of patients from the Hospital Viamed Santa Ángela de la Cruz was also considered in the subanalysis (Ferropenic, F). These patients had ferritin levels below 50 ng/mL and had been consecutively diagnosed with absolute iron deficiency (caused by excessive menstrual bleeding, or frequent blood donation) and did not present any active infection or malignancies. Local Ethical Committee from Virgen Macarena and Virgen del Rocío University Hospitals approved the study (PEIBA Acta CEI_03/2022 and CEI_04/2019).

### Laboratory measurements

Traditional iron-related biomarkers were determined in plasma samples of HIV, Young, and Elderly groups at the Biochemistry Service of HUVR and in ferropenic subjects at the Viamed Santa Ángela de la Cruz Hospital by the same standardized techniques. Briefly, total iron, transferrin, and soluble transferrin receptor (sTfR) were measured by photometry and ferritin by particle-enhanced immunoturbidimetric assay in a Hitachi Cobas C702 modular analyzer (Roche Diagnostics, Rotkreuz, Switzerland). Transferrin saturation index (TfSI) was estimated as (total plasma iron (µg/dL)x100)/(transferrin (mg/dL)x1.27).

In the case of ferropenic subjects (F), clinical data and values for such traditional iron-related parameters (total iron, transferrin, sTfR, and ferritin) had been measured following the standard protocols by Hospital Viamed Santa Ángela de la Cruz. For all samples, including those from ferropenic patients, plasma hepcidin was determined by ELISA (Hepcidin25 HS ELISA, DRG). Levels of the following inflammatory-related biomarkers were also measured (in all except for ferropenic subjects): high sensitivity C-reactive protein (hsCRP) and β2-microglobulin were determined by an immunoturbidimetric assay, homocysteine was determined by photometry; all of them were measured using Cobas 701 (Roche Diagnostics, Mannheim, Germany); D-dimers were determined by an automated latex enhanced immunoassay (HemosIL D-Dimer HS 500, Instrumentation Laboratory, Bedford, Massachusetts) and interleukin 6 (IL-6) was measured by ELISA (Human IL-6 Quantikine HS ELISA, R&D Systems) according to manufacturer’s instructions.

### Statistical analysis

Quantitative variables are all expressed as median and interquartile range [IQR]. Comparisons between groups were performed using the non-parametric Kruskal-Wallis test; when considered significant (*p*<0.05), multiple comparisons were performed using the nonparametric Mann-Whitney U-test. Categorical variables were recorded as the number of cases and percentages, with comparisons among groups using the χ2 or Fischer’s exact test. Associations between variables were explored by Spearman’s rho correlation coefficient and considered significant when *p*<0.05. Statistical analysis was performed in Statistical Package for the Social Sciences software (SPSS v21.0; IBM SPSS, Chicago, USA). Graphics were generated using GraphPad Prism (v8.0, GraphPad Software, Inc., USA).

## Results

### HIV-infected subjects exhibited altered levels of iron-related biomarkers compared to healthy controls

**Figure 1:**
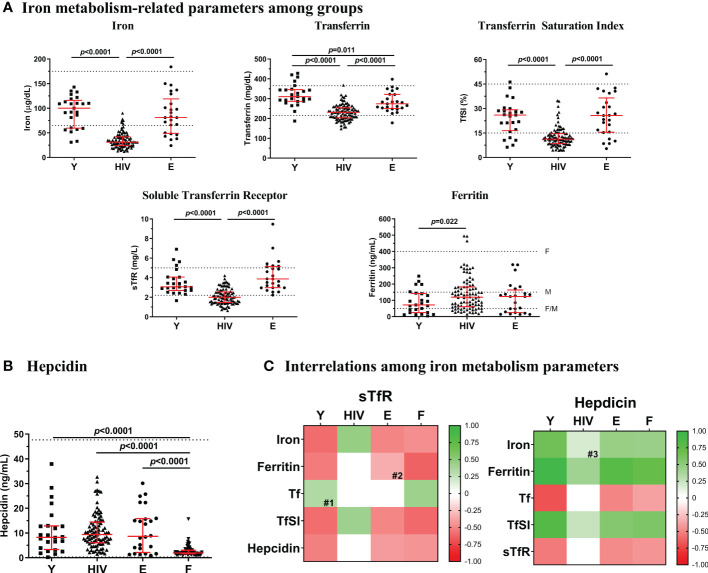
Iron-related parameters. **(A)** Levels of iron metabolism-related parameters (iron, transferrin, TfSI, sTfR and ferritin) in Young non-HIV (Y), HIV, and Elderly non-HIV € groups. **(B)** Hepcidin levels in Y, HIV, E and F (ferropenic group, with ferritin <50 ng/mL) groups. Comparisons were assessed using nonparametric Kruskal-Wallis/Mann–Whitney U tests. Variables with a *p*-value <0.05 were considered statistically significant. Grid lines represent minimum and maximum standard values; F, in females and M, in males. **(C)** Associations between sTfR or hepcidin and the rest of iron metabolism parameters in Y, HIV, E, and F groups. Color intensity of boxes represents Spearman’s rank correlation coefficient value as indicated in the color legend. All colored boxes represent statistically significant correlations, excepting for those noted as # (0.05 ≤ *p* ≤ 0.1). White boxes represent correlations with *p*-values >0.1. #1, p=0.092; #2, p=0.100; #3, p=0.096. Tf, transferrin; TfSI, transferrin saturation index; sTfR, soluble transferrin receptor.

Characteristics of the study subjects are summarized in **Table 1**. Men proportion was similarly higher in HIV-infected subjects (HIV; aged 42[34-47], 95% males) and Elderly (aged 82[78-88], 100% males) compared to Young (aged 40[30-58], 60% males). As expected, HIV showed lower CD4 T-cell counts (*p*=0.001) and higher CD8 T-cell counts (*p*<0.0001) compared to Young, therefore exhibiting a lower CD4/CD8 T-cell ratio (*p*<0.0001). Compared to Elderly, HIV showed similar CD4 T-cell counts, although higher CD8 T-cell counts (*p*<0.0001) and thus, lower CD4/CD8 T-cell ratio (*p*<0.0001). HIV showed lower levels of iron compared to Young and Elderly (31.0 [24.0-42.0] *vs*. 100.0 [59.5-116.0] and 81.0 [49.0-119.0] μg/dL, respectively, **Figure 1A**). Besides, 89.1% of HIV presented iron levels under 65 µg/dL (lower standard value), whilst only 28% of both Young and Elderly (*p*<0.0001). Transferrin was also significantly lower in HIV compared to healthy controls, although Elderly also showed lower levels compared to Young (HIV, 231.0 [203.3-253.8] *vs*. E, 274.0 [250.5-321.5] vs. Y, 310.0 [286.5-346.0] mg/dL). Accordingly, TfSI was also lower in HIV (HIV, 11.2 [8.5-14.6] *vs*. Y, 26.0 [16.5-29.3] and E, 25.7 [15.5-36.5] %) with 73.9% of the subjects under 15% (*vs*. 20% in both Young and Elderly, *p*<0.0001). Interestingly, sTfR was significantly lower in HIV compared to healthy controls (HIV, 2.00 [1.46-2.58] *vs*. Y, 3.07 [2.70-4.06] and E, 3.87 [2.98-5.15] mg/L), being 62% of the HIV under 2.2 mg/L (while only 4% and 0% in Young and Elderly, respectively; *p*<0.0001). However, we found higher ferritin levels in HIV compared to Young (119.0 [60.6-182.8] *vs*. 71.5 [24.8-142.2] ng/mL). Only 17.6% of HIV showed ferritin levels under 50 ng/mL, while 36% in both Young and Elderly (*p*=0.046).

**Table 1:** Characteristics of the study subjects.

Parameter	Young (N = 25)	HIV (N = 92)	Elderly (N = 25)	*p*(K-W)*	*p* (M-W;Y vs. HIV)	*p* (M-W;Y vs. E)	*p* (M-W;HIV vs E)
**Age (years)**	40 [30 – 58]	42 [34 – 47]	82 [78 – 88]	**<0.0001**	0.824	**<0.0001**	**<0.0001**
**Males, n (%)**	15(60)	87(95)	25(100)	**<0.0001**	**<0.0001**	**<0.0001**	0.234
**CD4 T-cells (cells/mm^3^)**	908 [789 – 1031]	660 [477 – 894]	625 [509 – 755]	**0.003**	**0.001**	**0.003**	0.972
**CD8 T-cells (cells/mm^3^)**	556 [340 – 727]	719 [602 – 1018]	341[182 – 436]	**<0.0001**	**<0.0001**	**0.004**	**<0.0001**
**CD4/CD8 T-cell ratio**	1.71 [1.34 – 2.35]	0.75 [0.56 – 1.33]	2.15 [1.50 – 3.09]	**<0.0001**	**<0.0001**	0.207	**<0.0001**
**CD4 T-cell nadir (cells/mm^3^)**	n/a	287 [177-392]	n/a				
**ART exposure (months)**	n/a	37 [26-39]	n/a				
**ART composition, n (%)**	n/a		n/a				
2NRTI + 1NNRTI		42 (45.7)					
2NRTI + 1PI		2 (2.2)					
2NRTI + 1II		12 (13.0)					
Other		6 (6.5)					
Unknown		30 (32.6)					

Quantitative variables are expressed as median [IQR], and the categorical variable “Males” is expressed as the number of cases (%). Variables with a p-value <0.05 were considered statistically significant, as shown in bold. K-W, nonparametric Kruskal-Wallis test; M-W, nonparametric Mann-Whitney U test; Y, Young; E, Elderly; ART, antiretroviral treatment; n/a, non-applicable; NRTI, Nucleoside Reverse Transcriptase Inhibitors; NNRTI, Non-Nucleoside Reverse Transcriptase Inhibitors; PI, Protease Inhibitors; II, Integrase Inhibitors. All the participants were from Europe, except for eight in the HIV group: 1 (1.1%) from Asia, 1 (1.1%) from Africa and 6 (6.5%) from South America. *For the qualitative variable “Males”, a chi-square test was applied.

### HIV-infected subjects showed similar hepcidin levels to healthy controls

No differences were found in the master regulator of the iron metabolism, hepcidin, between HIV, Young, and Elderly (**Figure 1B**). At this point, to explore whether a different context of iron deficiency could impact hepcidin levels, we additionally compared an external group of non-HIV subjects (N=84), with a diagnosed iron deficiency (Ferropenic, F), showing ferritin levels below 50 ng/mL [F; recently proposed as the optimum threshold for ID diagnose (15, 16)]. This group had a median of 42 [32-51] years old and included 80 (95%) females. Significantly lower levels of hepcidin were observed in Ferropenic when compared to the rest of the groups (**Figure 1B**).

**Figure 2:**
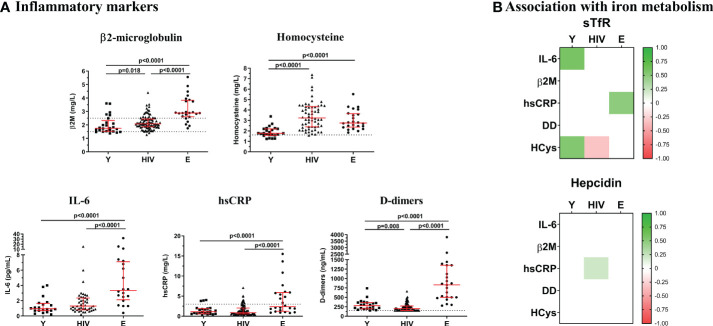
Inflammatory markers. **(A)** Levels of Β2-microglobulin, homocysteine, IL-6, hsCRP, and D-dimers were quantified in Young non-HIV (Y), HIV, and Elderly non-HIV (E) groups. Comparisons were assessed using nonparametric Kruskal-Wallis/Mann–Whitney U tests. Variables with a *p*-value <0.05 were considered statistically significant. Grid lines represent minimum and maximum standard values. **(B)** Associations between sTfR or hepcidin and inflammatory markers in Y, HIV and E groups. Color intensity boxes represents Spearman’s rank coefficient value as indicated in the color legend. All colored boxes represent statistically significant correlations. White boxes represent correlations with *p*-values >0.05. IL-6, interleukin 6; Β2M, Β2-microglobulin; hsCRP, high sensitive C reactive protein; DD, D-dimers; HCys, homocysteine.

We additionally characterized the inflammatory status in the study groups, as a potential modulator for hepcidin production, by measuring several representative biomarkers as shown in **Figure 2**. The highest levels of IL-6, Β2M, hsCRP, and D-dimers were found in Elderly. Compared to Young, HIV exhibited similar levels of IL-6 and hsCRP, but significantly higher levels of Β2M (2.08 [1.87-2.44] vs, 1.79 [1.60-2.12] mg/L; *p*=0.018) and homocysteine, being this last one even higher in HIV than in Elderly (HIV, 3.19 [2.36-4.32] vs. Y, 1.77 [1.52-2.23] vs, E, 2.62 [2.14-3.49] mg/L). Moreover, we observed two strong positive correlations between sTfR and IL-6 (*r*=0.585; *p*=0.009) and homocysteine (*r*=0.591; *p*=0.002) in Young that became lost or inverted respectively in HIV (IL-6, *r*=0.024, *p*=0.874; homocysteine, *r*=-0.321; *p*=0.012). Regarding hepcidin, no correlations were found in the Young group, whereas a positive association was observed with hsCRP in HIV (*r*=0.208; *p*=0.047).

### Normal associations between sTfR or hepcidin and traditional iron-related parameters were not present in HIV-infected subjects

We next explored potential interrelations between the sTfR or hepcidin and the traditionally tested parameters of iron homeostasis (**Figure 1C**). Interestingly, an association between two key elements of iron metabolism, hepcidin and sTfR was observed in both groups of healthy controls (Y, *r*=-0.516, *p*=0.010; E, *r*=-0.404, *p*=0.050). However, this negative association was not observed in HIV. Regarding sTfR, most of the associations found in Young were conserved in Elderly. However, in HIV not only the correlations between sTfR and iron or TfSI became positive (iron, *r*=0.451, *p*<0.0001; TfSI, *r*=0.415, *p*<0.0001) but also the association with ferritin was lost. Regarding associations with hepcidin, control groups showed positive correlations between iron and hepcidin (Y, *r*=0.594, *p*=0.002; E, *r*=0.502, *p*=0.013), which was only a trend in HIV.

Additionally, we checked all these interrelations in the non-HIV iron-deficient group (ferritin levels below 50 ng/mL; N=84), aiming to test whether the expected associations with hepcidin/sTfR were also altered under a non-infectious pathogenic condition (**Figure 1C**). Outstandingly, the correlations between sTfR or hepcidin and the rest of iron metabolism-related parameters were preserved in this group similarly as occurred in the non-pathogenic healthy controls.

**Figure 3:**
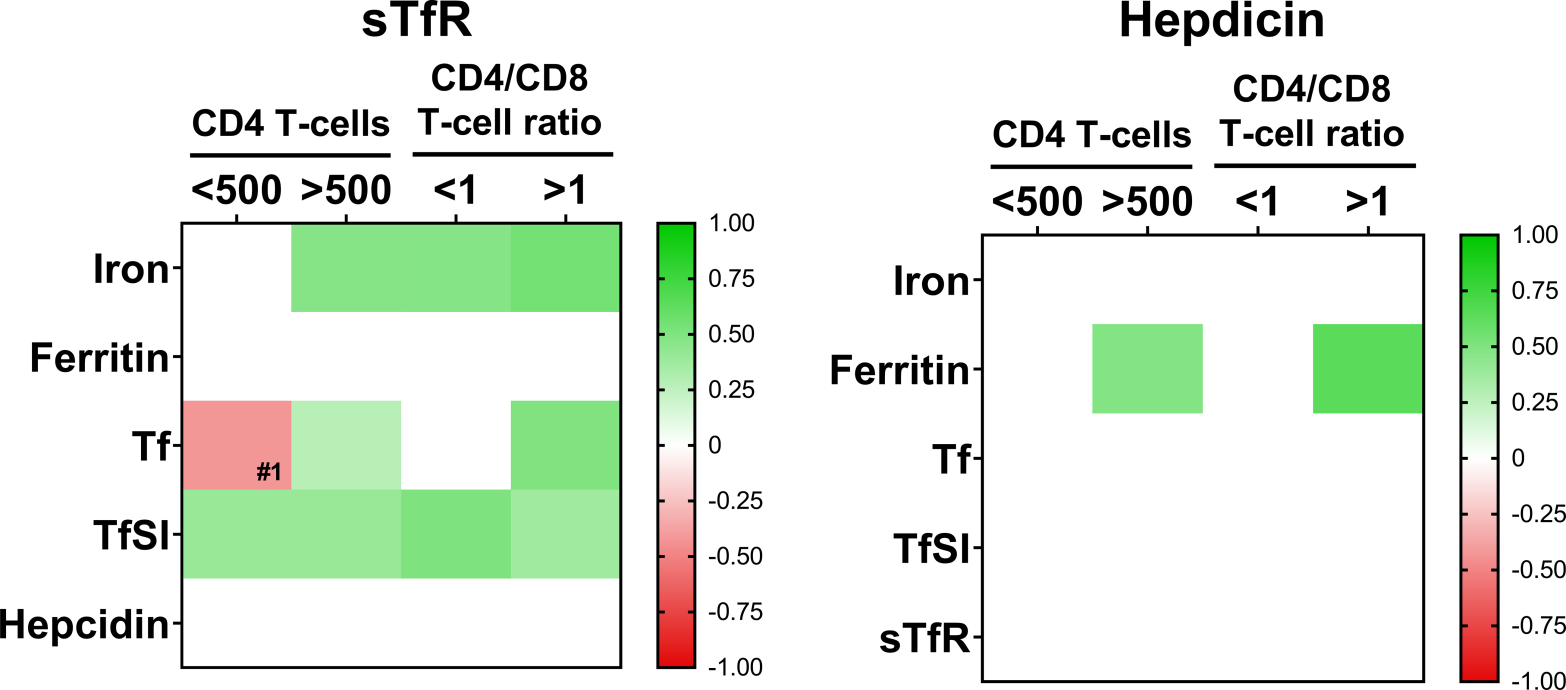
Interrelations among iron-metabolism parameters when patients are classified according to CD4 T-cell counts (below 500 cell/mm^3^, N=24 and over 500 cell/mm^3^, N=68) or CD4/CD8 T-cell ratio (ratio <1, N=55 and ratio >1; N=37). Color intensity of boxes represents Spearman’s rank correlation coefficient value as indicated in the color legend. All colored boxes represent statistically significant correlations, excepting for that noted as #1, *p*=0.072. White boxes represent correlations with *p*-values >0.1. Tf, transferrin; TfSI, transferrin saturation index; sTfR, soluble transferrin receptor.

Moreover, in order to check whether immunological reconstitution impacted on the interrelations between iron metabolism parameters, we analyzed the previous correlations classifying the patients according to CD4 T-cell counts (below/over 500 cells/mm^3^) or CD4/CD8 T-cell ratio (below/over 1). Interestingly, we observed that this loss of associations between parameters were more severe in immunological non-recovered patients, that is, CD4 T-cells below 500 and inverted ratio CD4/CD8 T-cell ratio (**Figure 3**).

Hemoglobin (Hb) values were only available for samples that had been collected in our Hospital (N=28). We observed that only 5 subjects (18%) were anemic (all males with Hb < 13 g/dL). The rest of the subjects (n=23) exhibited similar correlations as those observed in **Figure 1C** for the whole HIV group (**Figure S1**).

## Discussion

We report that virologically suppressed chronic HIV-infected subjects (under successful ART) exhibit altered levels of iron metabolism regulators that could underly a complex iron deficiency. Furthermore, the loss or even inversion of several relationships between sTfR or hepcidin and different iron-related parameters could suggest impaired iron homeostasis in this context. Outstandingly, in our population of chronically HIV-infected subjects, the levels of iron metabolism parameters are in accordance with a functional ID.

In our setting, only the diagnosed ID group showed decreased hepcidin levels whereas normal levels were observed in HIV. Few reports show hepcidin data in this context. A recent report showed lower hepcidin levels in chronically-treated HIV-infected subjects compared to healthy controls, however, such a control group showed abnormally high levels of hepcidin (17). In contrast, Armitage et al. (18) found increased hepcidin during the acute phase of HIV infection being its levels maintained during the chronic infection. Nevertheless, this study was carried out in patients with less than 2-year treatment. Importantly, Theurl et al. demonstrated that subjects in which anemia of inflammation (caused by chronic inflammatory diseases) and ID anemia coexisted, exhibited similar hepcidin levels to healthy subjects, while in absence of ID anemia increased levels of hepcidin were observed in subjects with anemia of inflammation (19).

Furthermore, we observed a loss or even inversion of the associations between iron metabolism parameters which could be denoting a disruption in the sTfR-hepcidin regulation in virologically suppressed chronic HIV-infected subjects, whilst such relationships seemed to be preserved in the ferropenic condition. This loss of associations was even more patent in immunologically non-recovered patients suggesting that these alterations could be directly associated to a poor immunological reconstitution. Even in high viral load treatment-naïve HIV-infected subjects in areas with a high burden of absolute ID probably caused by malnutrition, and where helminth and malaria infections are highly prevalent, the relationships between sTfR, hepcidin or ferritin were preserved in HIV-subjects in comparison to healthy controls (13).

Several parameters of inflammation, though not all, were found elevated in our chronic HIV population. Specifically, homocysteine has been reported to promote hepcidin secretion in cultured hepatocytes in a dose-dependent manner through the activation of BMP6/SAMD signaling (20). β2-microglobulin was also found elevated in HIV, being this molecule normally associated with HFE protein (Homeostatic Iron Regulator) in complexes that promote hepcidin production. Accordingly, previous studies have demonstrated that β2-microglobulin-deficient mice develop iron overload (21). Regarding other inflammatory biomarkers and, in accordance with our results, IL-6 and hsCRP have been found to normalize in HIV-infected subjects, after 96 weeks of suppressive ART (22). Furthermore, the inflammatory state found in the elderly is also in agreement with previous studies. Puzianowska-Kuznicka et al. (23) showed that IL-6 levels vary from 1.8-3.5 pg/mL (from 65-69-year-old persons to those over 90-year-old), while hsCRP varies between 2.2-2.5 mg/L in the same cohort (23).

**Figure 4:**
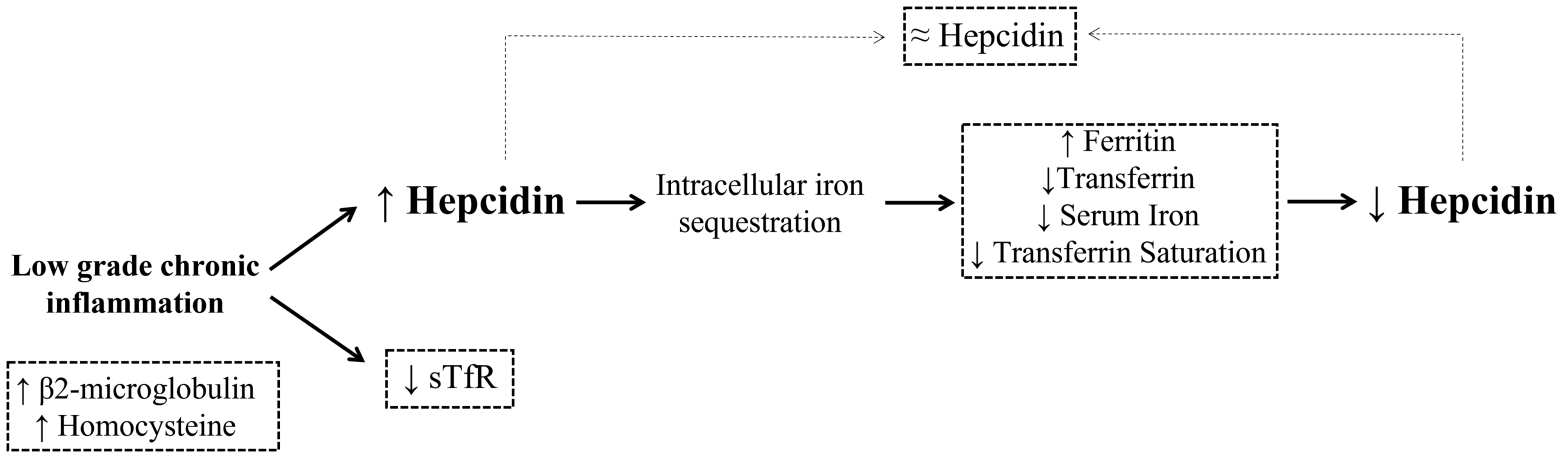
Proposed model of functional iron deficiency (ID) in virologically suppressed HIV-infected patients. Chronic HIV infection is known to cause a low grade inflammation that persists even after a successful ART. This setting is characterized by high levels of proinflammatory cytokines that promote hepcidin secretion, causing intracellular iron sequestration mainly in splenic macrophages and hepatic Kupffer cells by increasing stores (ferritin) and decreasing iron traffic (circulating iron, transferrin and transferrin saturation). As a result, hepcidin levels would decrease to favor again iron absorption from circulation, compensating then the elevated levels caused by the inflammatory environment. On the other hand, the decrease iron tissue demand during infection along with a likely mild bone marrow suppression would diminish the levels of sTfR. Altogether, inflammation would drive an impaired mobilization and utilization of the iron stores despite of proper requirements, therefore causing a functional ID in virologically suppressed chronic HIV infected patients. Grid lines represent results in our HIV cohort.

Based on these results, we hypothesize that a disruption in the sTfR-hepcidin regulation would not allow a proper balance in iron homeostasis. To our knowledge, this phenomenon has not been previously described in the setting of virologically suppressed chronic HIV-infected subjects. Inflammation not only would promote the decrease of iron tissue demand but could also cause iron redistribution *via* hepcidin secretion, which would cause intracellular iron sequestration, consequently adjusting hepcidin levels. Altogether, it would trigger an altered iron distribution and mobilization despite adequate iron storage, which supports a model of functional ID **(** **Figure 4** **)**. We are aware that this figure represents a global hypothesis derived from our results and that the implication of additional factors cannot be ruled out. In fact, it cannot be discarded that intestinal dysbiosis and enteropathy normally associated with chronic HIV infection [reviewed in (24)] could hinder intestinal iron absorption. Furthermore, defects in liver production, due to hepatic disorders, infection by hepatotropic viruses [highly prevalent in HIV-infected patients (25)] or hepatotoxicity (personal factors such as alcohol consumption, drugs, antibiotics, etc. or ART), all of them could potentially contribute to the negative regulation of hepcidin secretion.

This exploratory study has a main limitation: the gender differences between Young/Ferropenic and HIV/Elderly groups could be particularly relevant to iron metabolism regulation given the influence of sex hormones and other physiological processes such as the menstrual cycle. Indeed, some studies have reported higher levels of hepcidin in females than males, hindering iron absorption (26). However, the fact that iron regulation in the ferropenic group was preserved even though with such a high frequency of women compared to the HIV group, suggests that HIV infection weights more than sex hormones in this context. Other factors might also influence iron metabolism: weight and height (BMI), comorbidities, diet, antiretroviral treatment in the HIV group or polypharmacy in the elderly group, tobacco habit, viral hepatotropic infections…

Based on our results, we propose a hypothetical model of functional ID in virologically suppressed chronic HIV-infected subjects that could be contributing to their persistent immune dysfunction. This hypothesis needs further investigation including mechanistic approaches. Furthermore, interventions to address this complex condition could be interesting to be tested, however iron supplementation could result in useless. Indeed, a recent study demonstrated that iron supplementation in HIV-infected subjects with ID and/or anemia was associated with increased mortality (27). A reasonable approach to this complex setting could include the use of hepcidin antagonists, previously suggested to treat different iron-related disorders (28).

## Data availability statement

Raw data that support the findings of this study are available from corresponding author upon reasonable request.

## Ethics statement

The studies involving human participants were reviewed and approved by ethics committee of Virgen del Rocío and Virgen Macarena University Hospitals. The patients/participants provided their written informed consent to participate in this study.

## Author contributions

The author’s contributions were as follows, recruitment of subjects and sample/clinical data procurement (ML), experiments, and data collection (VG-R and AA-R), data analysis and interpretation (VG-R, AB-R, IO-M, MMP-B, ML, and YMP), manuscript preparation (VG-R and YMP). YMP conceived the study, obtained the funding, and supervised the project. All authors contributed to the article and approved the submitted version.

## Funding

This work was supported by grants from the Fondo de Investigación Sanitaria [FIS; PI18/01216 and PI21/00357] which is co-funded by Fondos Europeos para el Desarrollo Regional (FEDER) “Una manera de hacer Europa”, the Instituto de Salud Carlos III [FI19/00298 to VG-R, CM19/00051 to IO-M and CD19/00143 to AB-R], the Consejería de Transformación Económica, Industria, Conocimiento y Universidades [DOC_01646 to MP-B] and the Consejería de Salud y Familias of Junta de Andalucía through the “Nicolás Monardes” [C-0013-2017 to YMP]. The funders had no role in the study design, data collection and interpretation, or the decision to submit the work for publication.

## Acknowledgments

This study would have not been possible without the collaboration of all patients from Virgen del Rocío University Hospital and the residents from Santa Caridad Home for the Elderly, who consented to participate and the medical and nursery staff who have taken part in the project. We also thank Rafael Martínez Alba, director of the Santa Caridad Home for the Elderly, for allowing us to perform the study in his center and Juan Antonio Santamaría and Rafael Bernal for their clinical assistance with the resident included in this study. We also thank the participation of ferropenic patients and clinical staff from the Viamed Santa Ángela de la Cruz Hospital. We want to particularly acknowledge the patients in this study for their participation and to the HIV BioBank integrated in the Spanish AIDS Research Network and collaborating Centers for the generous gifts of clinical samples used in this work. The HIV BioBank, integrated in the Spanish AIDS Research Network, is supported by Instituto de Salud Carlos III, Spanish Health Ministry (Grant n° RD06/0006/0035, RD12/0017/0037 and RD16/0025/0019) as part of the Plan Nacional I+D+i and cofinanced by ISCIII- Subdirección General de Evaluación y el Fondo Europeo de Desarrollo Regional (FEDER)”. This study would not have been possible without the collaboration of all the patients, the medical and nursery staff and the data managers who have taken part in the project. The RIS Cohort (CoRIS) was funded by the Instituto de Salud Carlos III through the Red Temática de Investigación Cooperativa en SIDA (RIS C03/173, RD12/0017/0018 and RD16/0002/0006) as part of the Plan Nacional I+D+i and cofinanced by ISCIII-Subdirección General de Evaluación and the Fondo Europeo de Desarrollo Regional (FEDER).

## Conflict of interest

The authors declare that the research was conducted in the absence of any commercial or financial relationships that could be construed as a potential conflict of interest.

## Publisher’s note

All claims expressed in this article are solely those of the authors and do not necessarily represent those of their affiliated organizations, or those of the publisher, the editors and the reviewers. Any product that may be evaluated in this article, or claim that may be made by its manufacturer, is not guaranteed or endorsed by the publisher.
