# Supplementary material for: Dysregulation of iron metabolism modulators in virologically suppressed HIV-infected patients
Source: Front Immunol. 2022 Nov 25;13:977316. doi: 10.3389/fimmu.2022.977316 (PMC9732230; doi:10.3389/fimmu.2022.977316)
Supplement: Supplementary file 1 [file DataSheet_1.docx]

## Supplementary Material

## Figures


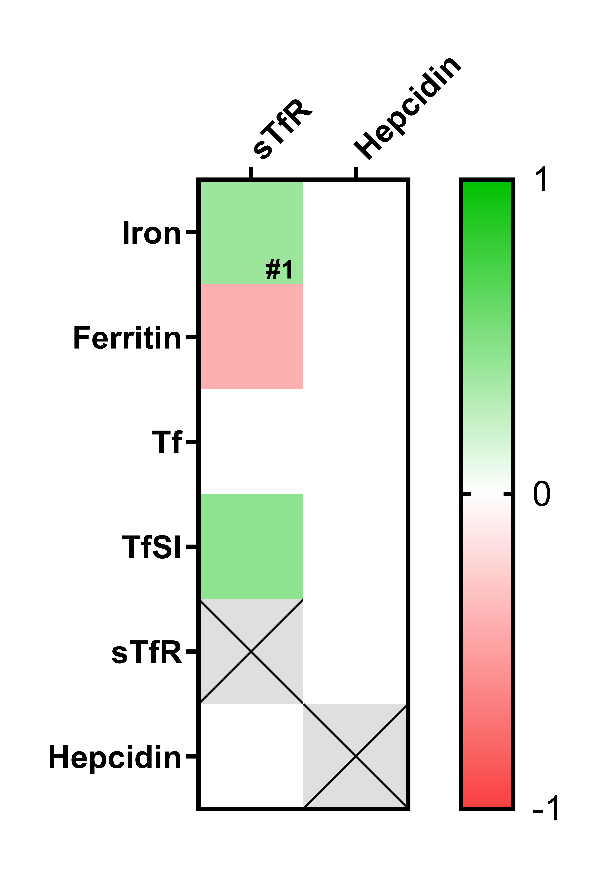


**Figure S1.** Associations between sTfR or hepcidin and the rest of iron metabolism parameters in non-anemic HIV-infected subjects (N=23). Color intensity of boxes represents Spearman's rank correlation coefficient value as indicated in the color legend. All colored boxes represent statistically significant correlations, excepting for that noted as #1 (*p*=0.092). White boxes represent correlations with *p*-values >0.1. Abbreviations: Tf, transferrin; TfSI, transferrin saturation index; sTfR, soluble transferrin receptor.
